# Sperm long non-coding RNAs as markers for ram fertility

**DOI:** 10.3389/fvets.2024.1337939

**Published:** 2024-05-10

**Authors:** Mustafa Hitit, Abdullah Kaya, Erdogan Memili

**Affiliations:** ^1^Department of Genetics, Faculty of Veterinary Medicine, Kastamonu University, Kastamonu, Türkiye; ^2^College of Agriculture, Food and Natural Resources, Cooperative Agricultural Research Center, Prairie View A&M University, Prairie View, TX, United States; ^3^Department of Animal and Dairy Sciences, College of Agricultural and Life Sciences, University of Wisconsin–Madison, Madison, WI, United States

**Keywords:** ram, sperm, fertility, long non-coding RNAs, gene expression

## Abstract

It is critical in sheep farming to accurately estimate ram fertility for maintaining reproductive effectiveness and for production profitability. However, there is currently a lack of reliable biomarkers to estimate semen quality and ram fertility, which is hindering advances in animal science and technology. The objective of this study was to uncover long non-coding RNAs (lncRNAs) in sperm from rams with distinct fertility phenotypes. Mature rams were allocated into two groups: high and low fertility (HF; *n* = 31; 94.5 ± 2.8%, LF; *n* = 25; 83.1 ± 5.73%; *P* = 0.028) according to the pregnancy rates sired by the rams (average pregnancy rate; 89.4 ± 7.2%). Total RNAs were isolated from sperm of the highest- and lowest-fertility rams (*n* = 4, pregnancy rate; 99.2 ± 1.6%, and 73.6 ± 4.4%, respectively) followed by next-generation sequencing of the transcripts. We uncovered 11,209 lncRNAs from the sperm of rams with HF and LF. In comparison to each other, there were 93 differentially expressed (DE) lncRNAs in sperm from the two distinct fertility phenotypes. Of these, 141 mRNAs were upregulated and 134 were downregulated between HF and LF, respectively. Genes commonly enriched for 9 + 2 motile cilium and sperm flagellum were *ABHD2, AK1, CABS1, ROPN1, SEPTIN2, SLIRP*, and *TEKT3*. Moreover, *CABS1, CCDC39, CFAP97D1, ROPN1, SLIRP, TEKT3*, and *TTC12* were commonly enriched in flagellated sperm motility and sperm motility. Differentially expressed mRNAs were enriched in the top 16 KEGG pathways. Targets of the differentially expressed lncRNAs elucidate functions in *cis* and *trans* manner using the genetic context of the lncRNA locus, and lncRNA sequences revealed 471 mRNAs targets of 10 lncRNAs. This study illustrates the existence of potential lncRNA biomarkers that can be implemented in analyzing the quality of ram sperm and determining the sperm fertility and is used in breeding soundness exams for precision livestock farming to ensure food security on a global scale.

## 1 Introduction

Male fertility is crucial for animal reproduction as it encompasses the successful fertilization of an egg with a viable spermatozoon, subsequently leading to embryonic and fetal development, ensuring the species' continued existence. Fertility is an economically important trait, and ram flock represents more than 50% of the genetics of every sheep farmer's flock (1). Thus, proper management of rams for maximum performance and longevity is vital to the success and reproductive efficiency of sheep farming (2). Accordingly, advancing ram fertility in the livestock system is imperative to provide animal-based food demands to feed the ever-increasing human population on a global scale.

In addition to genetic evaluation and testing, rams have been selected according to breeding soundness exam (BSE), which requires a series of examinations, including physical exam, scrotal measurement, sperm morphology, and motility. Despite massive attempts to evaluate ram fertility using the BSE, the predictability of ram fertility still awaits improvement. Non-compensable factors, such as DNA damage, RNA molecules (3), and protein markers (4, 5) in sperm fertility, are attributed to minute sperm abnormalities that cannot be determined using conventional procedures (6). Advanced omics approaches have paved the way for underlying molecular mechanisms related to spermatogenesis, fertilization, and embryogenesis (7, 8). Such methods may be used more widely in the future for producing farm animals in combination with the evaluation of semen parameters (9, 10), while also estimating accurate sperm fertility markers in farm animals (11, 12).

Spermatozoa can transmit not just the paternal DNA but also certain RNA molecules and transcription factors, presumably inherited into the oocytes during fertilization (13). Researchers have recently used cutting-edge approaches for discovering small noncoding RNAs with various nucleotide lengths and biogenesis processes, such as PIWI-interacting RNAs (piRNA), microRNAs (miRNA), tRNAs, and long noncoding RNA (lncRNA)-derived short RNAs (14–17). Sperm bearing RNA molecules are implicated in spermatogenesis and embryo development at transcriptional and posttranscriptional levels (18–20). As such, they play roles in regulating spermatogenesis and fertilization by transferring small noncoding RNA (sncRNA) and lncRNA into the oocyte (3, 19, 21). In addition, paternally derived noncoding RNAs are key regulators of preimplantation embryos (22) since some of them are involved in the control of gene expression in zygotic and early embryonic development (23). Accordingly, sperm noncoding RNAs can enhance the transmission of epigenetic information to the offspring (13) because environmental and metabolic-induced modifications of sperm cells may influence the epigenetic modulation of embryo development by changing the gene expression through noncoding RNAs (24–27).

LncRNAs comprise nucleotides located in the cytoplasm and nucleus, which are transcribed by RNA polymerase II and are longer than 200 nucleotides and lack protein-coding capacity. LncRNAs, with their higher and more stable structures, modulate gene expression (*cis* and *trans* manner) at several levels, including epigenetic, transcriptional, posttranscriptional, and posttranslational, through their interactions with mRNA, proteins, and other sncRNAs. They have significant functions in biological processes such as modifying the chromatin structure, activating gene expression, inhibiting gene expression, and translating mRNA molecules. LncRNAs have crucial roles in regulating the many biological processes that are highly expressed in bovine and mouse testis and mature sperm (28, 29). There is a linkage between noncoding RNA markers and male fertility, as the transcripts can be involved in the prediction of fertility (30, 31). The lncRNAs differentially expressed in distinct motility phenotypes of bovines and humans (32) imply that sperm lncRNA may possess functional roles in fertility (33). Using RNA sequencing technologies, sperm bearing RNA was found to be conversed among many species, such as stallions, goats, and boar (34–36). The unique male sperm ncRNAs with consistent fertility phenotypes can be valuable as potent fertility biomarkers. Accordingly, this study aimed to uncover long noncoding RNA profiles from ram sperm possessing distinct fertility phenotypes.

## 2 Material and methods

### 2.1 Ram fertility assessment and experimental design

The Republic of Turkey's Ministry of Agriculture and Forestry's Institute of Bahri-Dagdaş International Agricultural Research supplied information on the fertility phenotypes of adult rams. Pregnancy rates from natural mating were used to determine the fertility of mature rams (*n* = 66) at least 3–4 years old during the breeding seasons of 2017–2018–2019. The ewes' estrus was detected using teaser rams, who were not permitted to mate, by covering the prepuce area. The teaser rams were presented to the ewes early in the morning for about 30 min. The estrus was considered to be ewes seeking, standing for teasing, and allowing mount attempts by teaser rams. A handler selected estrous ewes and brought them into an enclosure along with a randomly selected single ram for natural mating. Throughout the breeding season, estrus detection was maintained, and ewes were accepted to mate with the chosen ram at random. Ewes were regarded as pregnant if they did not return to estrus within 35 days of mating. In addition, the number of pregnant and non-pregnant ewes for each ram was confirmed by matching the mating and lambing dates according to the duration of pregnancies. The rams' fertility scores were rated based on their conception rates. The rams were split into two groups based on their fertility levels: the high-fertility (HF) group (*n* = 31; 94.5 ± 2.8%) and the low-fertility (LF) group (*n* = 25; 83.1 ± 5.73%). The average pregnancy rate was 89.4 ± 7.2% (*n* = 66), and the rams were divided into these groups based on their fertility levels. We conducted an *a priori* power analysis using G^*^Power3 (V3.1.9.4) to test the differences between two independent group means using a two-tailed test, an effect size (*d* = 2.52), and an alpha level of .05. The result showed that a total sample of eight animals with two equal-sized groups of *n* = 4 was required to achieve a power of .80 for RNA profiling. However, we excluded the rams that did not have 50 mating and were not used, so we profiled four animals for each group out of a total of 56. During the breeding season, each ram served at least 50 ewes in both groups.

### 2.2 Semen collection

The Bahri-Dagdaş Research Center Ethical Committee, Turkey (Number: 22.12.2016/58), approved the animal procedures. We trained the rams to obtain sperm using an artificial vagina (AV) that enabled them to mount on teaser ewes during estrus. Rams were permitted to ejaculate into the AV upon mounting. The first three collections were discarded prior to the collection of research samples, which was followed by semen collection and processing for use in research. Fertility rates that were 1 standard deviation above or below the mean were termed outliers. Four rams with the highest fertility (pregnancy rate; % 99.2 ± 1.6) and four rams with the lowest fertility (pregnancy rate; % 73.6 ± 4.4) were selected for lncRNA sequencing with high confidence. Subsequently, we collected about 2 × 10^9^/ml spermatozoa per ejaculate, and then the aliquots from each sample were adjusted to a final concentration of 10^7^/ml in straws and frozen at −80°C until lncRNA analysis.

### 2.3 RNA isolation

Prior to RNA isolation, we purified sperm by filtering the semen samples with a 500-mesh sieve to eliminate cell debris. Then, samples were treated with a somatic cell lysis solution (0.3% Triton X-100 and 0.1% SDS in DEPC-treated H_2_O) for 30 min on ice to eradicate somatic cells, followed by microscopic analysis of non-sperm cell contamination. We isolated total RNA from the purified ram sperm (*n* = 4, for each group) samples using the SanPrep column microRNA miniprep kit (Bio Basic Inc, Canada) with slight modification using the manufacturer's protocols. We added 800 μl of a guanidine–thiocyanate lysis buffer enriched in 20 mM DL-dithiothreitol onto the pellet, and then sperm cells were homogenized by passing the samples through a 26-G needle syringe 20–25 times. After other contaminants were thoroughly removed and total RNA was attached to the membrane, an on-column DNase digestion was carried out to remove any traces of DNA contamination. We evaluated the concentration and integrity of the total RNA samples using a NanoDrop (Colibri Microvolume Spectrometer, Titertek-Berthold, Germany) and a 2100-Bioanalyzer with the RNA 2100 Nano Chip (Applied Biosystems, Carlsbad, CA, USA), respectively.

### 2.4 Library preparation for lncRNA sequencing

Each RNA sample was utilized to prepare 2 g of RNA for the RNA library, and ribosomal RNA was first eliminated using the Ribo-ZeroTM rRNA Removal Kit from Epicenter. Then, using the NEBNext^®^ UltraTM-Directional RNA Library Prep Kit from Illumina^®^ (NEB, USA) in compliance with the commercial kit protocol, sequencing libraries were developed utilizing the rRNA-depleted RNA. Reverse transcription was used to create the first strand of the cDNA following fragmentation with an average length of 200 bp. The Agilent Bioanalyzer 2100 equipment was used to evaluate library quality following product purification using the AMPure XP system. As a result, libraries underwent sequencing using the Illumina NovaSeq 6000 (Illumina Inc., San Diego, CA, United States), and Novogene Corporation (Beijing, China) generated 150-bp paired-end reads.

### 2.5 RNA-Seq read alignment and transcript assembly

Initially, rRNA, adapter sequences, empty reads, and low-quality reads were eliminated from the raw data. All trimmed reads were confirmed to satisfy the quality threshold (*Q*-score; Q20 and Q30) to ensure that there was no bias in the evaluation step toward approaches that favor maximum read. The Phred scale indicating the reliability of base-calling, with Q20 representing a base call accuracy of 99% (or a 1% chance of error) and Q30 representing a base call accuracy of 99.9% (or a 0.1% chance of error) was used as the quality score. The Ovis aries (v4.0) reference genome was indexed with Bowtie v2.0.6, and the processed paired-end reads were mapped to that genome with HISAT2 2.1.0 (37). Each sample's mapped reads were constructed using StringTie (v1.3.1) (38). Finally, Cuffcompare, a program included in Cufflinks (v2.1.1), was used to annotate the assembled transcripts.

### 2.6 Putative lncRNA identification

To classify newly screened lncRNAs with respect to their positional relationship with known mRNAs, putative lncRNAs were identified. To minimize the false-positive rate (FDR), assembled transcripts were classified to retrieve putative lncRNAs, such as lincRNA, antisense lncRNA, intronic lncRNA, and sense-overlapping. (A) Transcripts with a single exon were eliminated. (B) Transcripts with fewer than 200 nucleotides were eliminated. (C) Using Cufflinks v2.1.1, the annotated lncRNAs in the database were used to exclude the transcripts that overlapped with the exon region of the database annotation. (D) All transcripts with modest levels of expression [FPKM thresholds were set for the categorization of transcript expression levels; genes with very low or no expression (FPKM < 0.5), and FPKM = 0.5 was chosen as the cutoff to filter out the average read coverage per transcript which was much higher than the other transcript] were omitted (FPKM of a single exon transcript). Using three methods, namely, Coding-Non-Coding-Index (CNCI) (39), Pfam-scan (40), and coded potential calculator (CPC) (41), all estimated transcripts with coding potential were filtered out, and a set of putative lncRNAs was compiled from those with noncoding potential. Using Cuffcompare, the distinct categories of lncRNAs were obtained.

### 2.7 Analysis of mRNA and lncRNA expression levels

The FPKM value was used to evaluate levels of mRNA and lncRNA expression. Cuffdiff (v2.1.1) was used to determine lncRNA FPKM values. Later, the statistically significant DE genes were quantified by a log_2_-fold change higher or equal to 2 (*P*-value < 0.05) or *P*-adjust < 0.05 (applied correction for multiple testing to the *P*-values to FDR), using Ballgown (42).

### 2.8 Target gene prediction

DE lncRNAs were selected to determine target genes. Pearson's correlation was used to assess potential coexpression between lncRNAs and mRNAs. A Pearson correlation >0.7 and a *P*-value of 0.05 were used to determine a positive association between an lncRNA and an mRNA. LncRNAs can act as *cis* regulators by remodeling factors onto local chromatin. We described *cis*-modulated genes as protein-coding genes that were coexpressed with one dysregulated lncRNA and were within 30 kb upstream or downstream in genomic distance in the same allele. To participate in certain biological processes, key transcription factors (TFs) are regulated in a *trans* manner by unique lncRNAs. As a result, we matched these lncRNAs' coexpressed mRNAs to mRNAs that were regulatory targets of specific TFs to anticipate that these lncRNAs might be involved in pathways controlled by these TFs.

### 2.9 Gene ontology terms and KEGG pathway enrichment

We assessed DE mRNAs for gene ontology (GO) enrichment analysis using g:Profiler (43). GO terms possessing a corrected *P*-value < 0.05 were accepted as significantly enriched by DE genes. The statistical enrichment of lncRNA target genes was examined for KEGG pathway functional analysis based on the reactome pathway database by WebGestalt (WEB-based Gene SeT AnaLysis Toolkit) with *P* < 0.05 and FDR < 5.0% (44). The following parameters were specified for the enrichment analysis. A particular organism was labeled as *Ovis aries* (sheep). Sequential GO analyses (biological process; BP, cellular component; CC, and molecular function; MF) were conducted. The g:SCS method is used to compute multiple testing corrections for *P*-values based on GO and pathway enrichment analysis, with the user threshold set at 0.05.

### 2.10 Statistical analysis

Data were analyzed through SPSS software (version 22.0). Statistical plots were generated using GraphPad Prism 9 (GraphPad Software, USA). During the experimental process, four biological replicates were included with the measurement repeated twice. LF vs. HF groups were analyzed using an independent *t*-test. Significance was accepted at *P*-value = 0.05.

## 3 Results

### 3.1 Overview of sequencing data in ram sperm between LF and HF

In the current study, we pooled two cDNA libraries out of the eight total RNAs (LF and HF, each = 4) obtained from low- and high-fertility ram sperm. The total number of raw readings obtained from all cDNA libraries was 91,108,174. Upon filtering the reads, a total of 89,252,823 clean reads were obtained. The Q20 (%) percentages were 96.06 and 95.39 for LF and HF, respectively. The Q30 (%) percentages were shown to be 90.96 and 89.50 for LF and HF, respectively. The percentages of GC content (%) were shown to be LF, 60.89, and HF, 62.37 (Table 1). Furthermore, after aligning the clean reads with the ovine reference genome through the TopHat2 algorithm, we discovered that the total mapped reads or fragments referring to all samples exceeded 75% and were mapped in the reference genome (Table 2).

**Table 1:** Summary of data production.

**Sample**	**Raw_reads**	**Clean_reads**	**Raw_data (G)**	**Clean_data (G)**	**Error_rate (%)**	**Q20 (%)**	**Q30 (%)**	**GC_ content (%)**
LF	41,020,716	40,183,273	12.3	12.1	0.03	96.06	90.96	60.89
HF	50,087,458	49,069,550	15	14.7	0.03	95.39	89.5	62.37

Raw read statistics: the total number of reads for each file is determined, and each set of four consecutive lines represents the information for one read. Clean reads are the same as raw reads, but only the filtered reads are used to calculate the results of any further study; Clean reads (G) the sum of a sequence's length and number, expressed in giga bases; Error rate: the sequencing error rate; Q20, Q30: the proportion of all bases for which the Phred score is 20 or 30 or above, indicating base call accuracy; the proportion of guanine (G) and cytosine (C) in all bases is known as GC content.

**Table 2:** A list of reads that were mapped to the reference genome.

**Sample name**	**HF**	**LF**
Total reads	98,139,100	80,366,546
Total mapped	82,508,774 (84.07%)	69,017,041 (85.88%)
Multiple mapped	3,526,455 (3.59%)	2,394,038 (2.98%)
Uniquely mapped	78,982,319 (80.48%)	66,623,003 (82.90%)
Read-1	40,351,963 (41.12%)	33,802,488 (42.06%)
Read-2	38,630,356 (39.36%)	32,820,515 (40.84%)
Reads map to “+”	39,407,126 (40.15%)	33,255,875 (41.38%)
Reads map to “–”	39,575,193 (40.33%)	33,367,128 (41.52%)
Non-splice reads	77,745,931 (79.22%)	65,903,933 (82.00%)
Splice reads	1,236,388 (1.26%)	719,070 (0.89%)

Total reads: the number of reads after data filtration (clean data); Total mapped represents the total quantity of mappable reads. If a suitable reference genome is available and no contamination occurs during the experimental procedure, the percentage will typically exceed 70%. Numerous mappings: the number of sequences mapped to multiple reference sequence positions; specifically mapped: the number of reads that map to a particular position in the reference sequences. Map to “+”; Reads that map to “–”: the number of reads that are mapped to the minus strand. Quantity of reads mapped to two exons; also known as junction reads. Similarly, non-splice reads are those that are completely mapped to a single exon. The ratio of splice readings to total read length is proportional.

### 3.2 Chromosome read distribution and known RNA types

The distribution intensity of the total mapped reads was split up and computed for both the plus and minus strands within each chromosome (Supplementary Table 1). The distributions of reads in the known RNA types for LF and HF are depicted in Table 3. The corresponding data related to these distributions can be found in Supplementary Table 2.

**Table 3:** A list of reads that were mapped to the reference genome.

**Classification of mapped reads (LF)**	**Classification of mapped reads (HF)**
Others (49,390,745 [78.4%])	Others (57,960,150 [77.95%])
Protein_coding (12,785,367 [20.3%])	Protein_coding (15,352,804 [20.65%])
lncRNA (697,609 [1.1%])	lncRNA (855,897 [1.15%]
pseudogene (125,827 [0.2%])	pseudogene (137,580 [0.2%])
rRNA (10,395 [0.02%])	rRNA (11,500 [0.02%])
miRNA (5,685 [0.01%])	miRNA (7,407 [0.01%])
misc_RNA (3,588 [0.0%])	misc_RNA (13,778 [0.0%])
ribozyme (2,495 [0.0%])	ribozyme (7,883 [0.0%])

Distribution of mapped reads in known types of RNAs for low fertility (LF) and high fertility (HF).

### 3.3 Novel lncRNA identification in ram sperm between LF and HF

**Figure 1:**
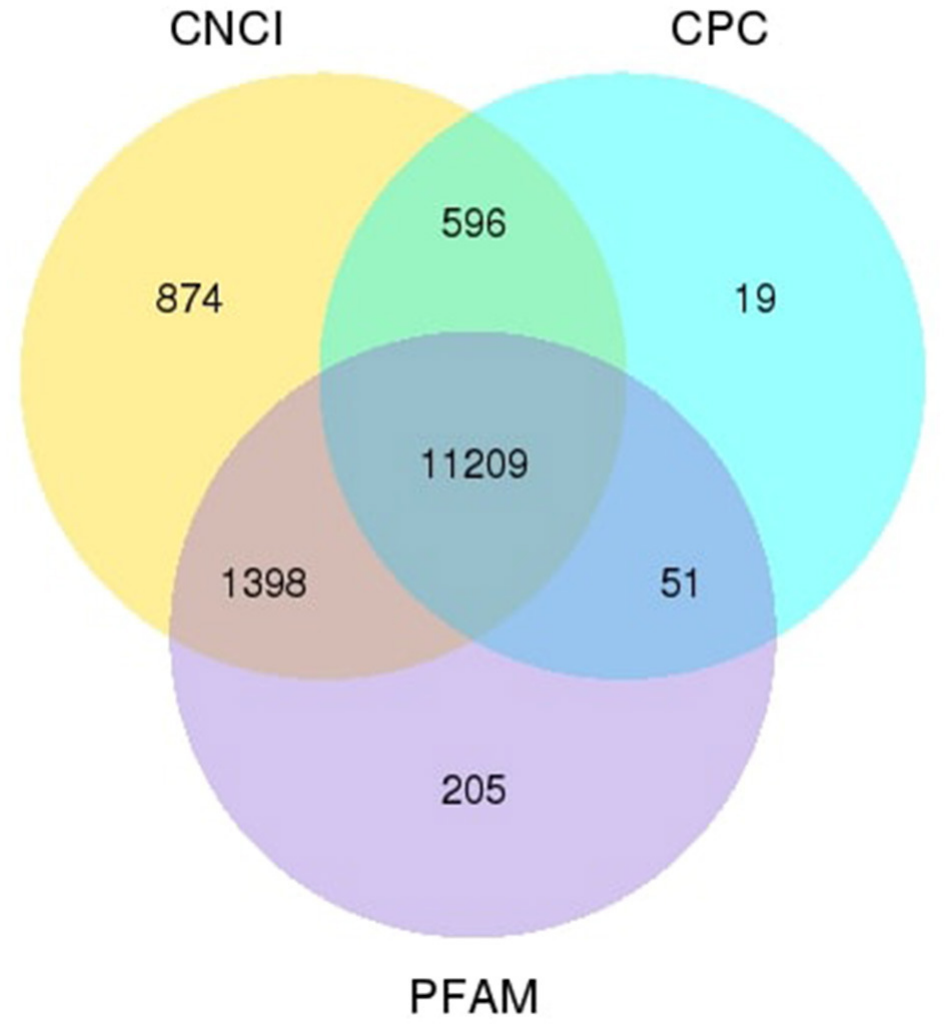
Venn diagram. The numerical values within each circle and the areas of overlap symbolize the total count and the number of noncoding transcripts that are found out by the software.

We applied the lncRNA filtering method to determine the novel lncRNA candidates in ram sperm. The specific filtering technique is outlined in Supplementary Table 3. Using this lncRNA filtering process, we identified a total of 14,352 lncRNAs, of which 11,209 were novel candidates, the data for which are supplied in Supplementary Table 4. We utilized three distinct software packages (CNCI, CPC, and Pfam) to estimate the potential protein-coding ability of the transcripts (Figure 1).

### 3.4 Identification and characteristics of lncRNAs between LF and HF in ram sperm

**Figure 2:**
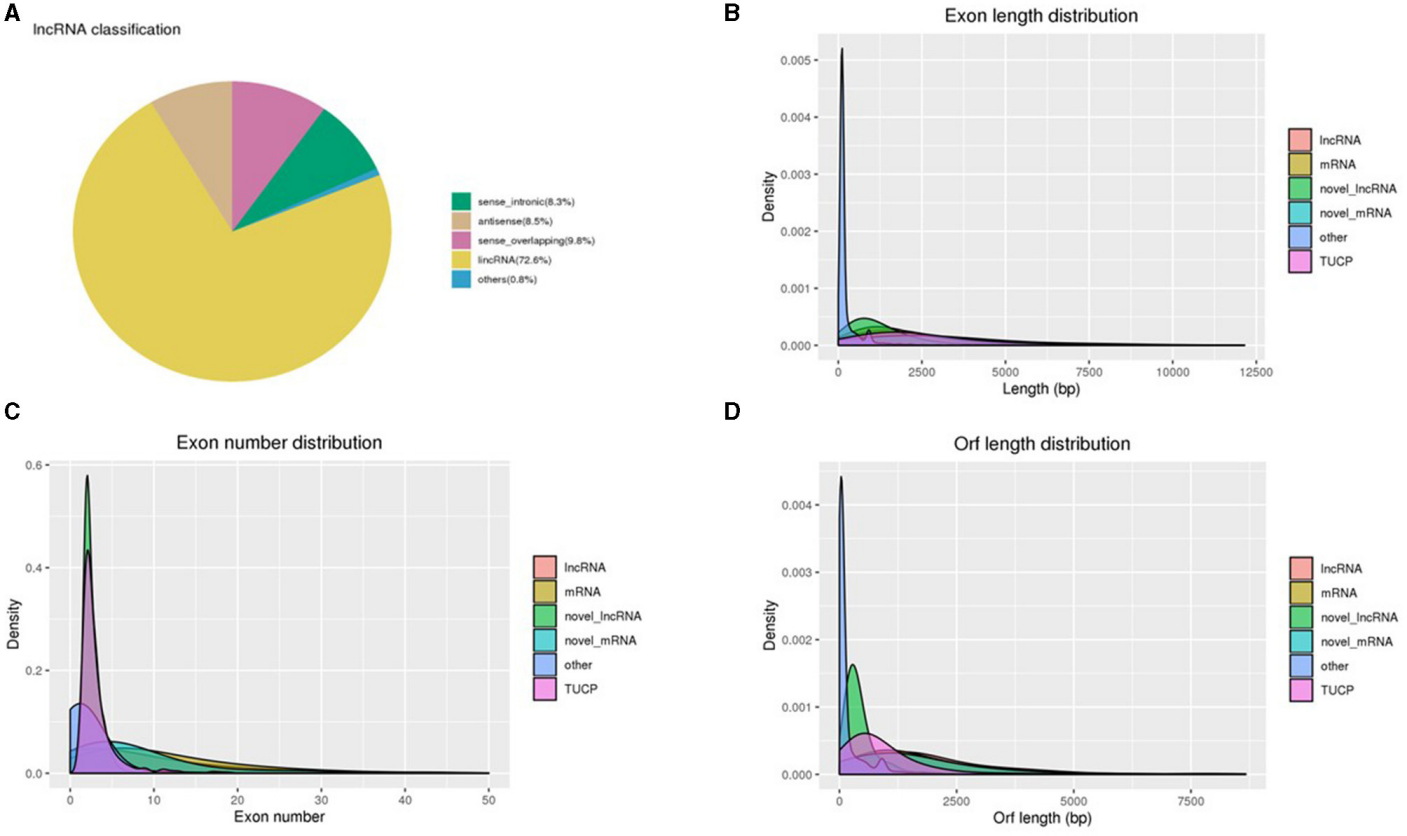
**(A)** Distribution of various lncRNA types. **(B)** Comparison of the lengths of lncRNAs, mRNAs, novel lncRNA–mRNA, and TUCP. **(C)** A comparison of the exon counts of lncRNAs and mRNAs. **(D)** A comparison of the ORF lengths of lncRNAs and mRNAs.

The primary characteristics of the lncRNAs' biotype distribution and length, exon intensity, and ORF length were examined as a consequence of the sequence analysis. Using Cufflinks, we divided lncRNAs into three groups: long intervening noncoding RNA (lincRNA), antisense lncRNA, and intronic lncRNA. Of these, 8.3%, 8.5%, 9.8%, 72.6%, and 0.8% of the attained lncRNAs were sense intronic, antisense, sense overlapping, lincRNA, and others, respectively (Figure 2A). Using this classification scheme, we revealed that the majority of the sperm-specific lncRNAs (72.6%) were lincRNAs. The exon lengths of the obtained lncRNAs were between 126 and 20,040 bp, and approximately 37% of the lncRNAs were demonstrated to be intense in 946–1,356 bp length (Figure 2B), with 1,542 bp being the median value. The exon length of the obtained mRNAs ranged between 75 and 26,726 bp, with around 10% of the mRNAs being intense at 835–1,025 bp (Figure 2B). Furthermore, lncRNAs have been shown to be more abundant in the first and fourth exons, whereas mRNAs were found to be more abundant in the first and seventh exons (Figure 2C). The length of the ORF of the lncRNAs was between 54 and 20,037 bp, with a median value of 1,461 bp (Figure 2D). For mRNAs, the ORF length was reported as 3–26,460, and the median value was 1,332.

**Figure 3:**
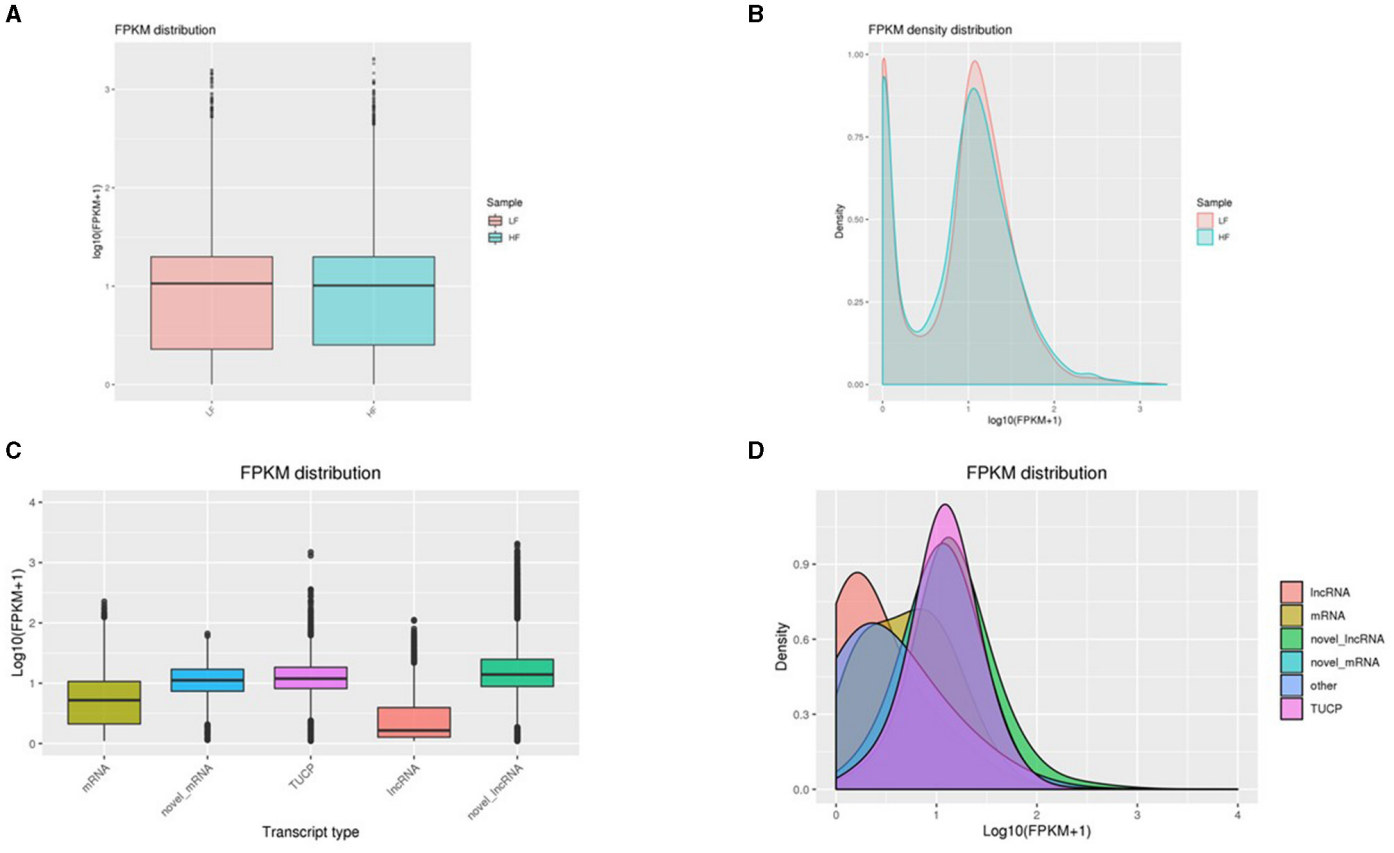
**(A, B)** A comparison of gene expressions. Box plot depicting FPKM values. The *X* and *Y* axes reflect the corresponding sample name and log_10_(FPKM + 1) value, respectively. For each sample, the plot region reflects, from top to bottom, the maximum, upper quartile, median, lower quartile, and minimum statistics. (2) Density distribution of Fragments Per Kilobase of transcript per Million mapped reads. The *X* and *Y* axes show, respectively, the value of log_10_(FPKM + 1) and the density of genes. **(C, D)** Violin plot for distinct forms of lncRNA, mRNA, and TUCP transcription. The *X*-axis of the FPKM violin plot displays the sample names, while the *Y*-axis displays the log_10_(FPKM + 1). Each violin plot possesses five statistical parameters (max value, upper quartile, median, lower quartile, and min value). The breadth of the violin plot indicates gene density.

The expressions associated with transcripts of lncRNA, mRNA, novel lncRNAs, and TUCP were evaluated using Cuffdiff. The most common approach for predicting gene expression levels, FPKM, is based on the effects of gene length on sequence depth and fragments described in the RNA sequence. The FPKM values associated with samples were calculated (Supplementary Table 5). Upon comparing FPKM values between LF and HF, no significant change was observed (Figures 3A, B). Moreover, the expression levels related to novel lncRNAs were the highest among the transcripts. TUCP and novel mRNA transcripts were greater than those of lncRNA and mRNAs, while lncRNA transcripts were similar to those of mRNA (Figures 3C, D).

### 3.5 LncRNAs and mRNAs that are differentially expressed in LF and HF

**Figure 4:**
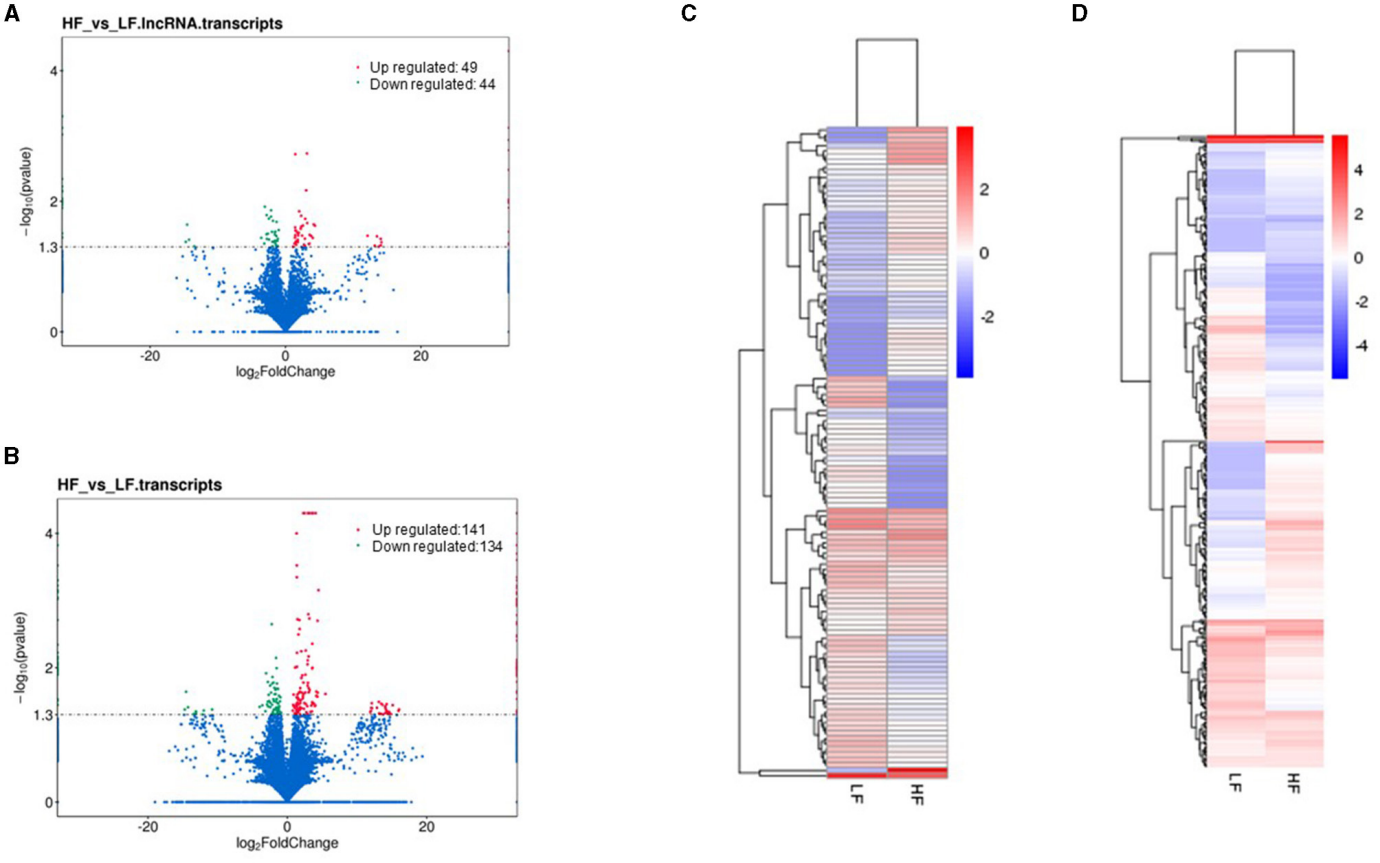
Volcano plot: **(A, B)** show differentially expressed lncRNAs and mRNAs in sperm between LF and GF rams, respectively. Heat map of clustering of genes; **(C, D)** show differentially expressed mRNAs and lncRNAs between LF and HF ram sperm (fold change >2, *p* < 0.05), respectively. Colors indicate the following red; up and blue; down.

Cuffdiff was employed to identify differentially expressed lncRNAs, mRNAs, and TUCPs. As a result, in HF and LF ram sperm, 93 lncRNAs and 275 mRNAs were reported to be differentially expressed (DE). We discovered 49 lncRNAs that are significantly upregulated and 44 that are downregulated between LF and HF groups. In addition, we discovered that 141 mRNAs were upregulated, whereas 134 were downregulated. Volcano plots show the up and downregulation highlights (Figures 4A, B; Supplementary Table 6). We also examined the expression patterns of DE lncRNAs and mRNAs using hierarchical clustering analysis, which serves as an additional approach for shedding light on differentially expressed genes by grouping genes with comparable expression patterns. The transcript FPKMs were utilized for hierarchical clustering, with discrete colors indicating the direction of the expression level. The clustering of genes on the left was caused by similar expressions (fold change >2, *P* < 0.05) and LF and HF ram sperm, whereas the expression change from blue to red indicated them as increasingly increased (Figures 4C, D).

### 3.6 DE mRNA functional annotation and KEGG pathways analysis

**Figure 5:**
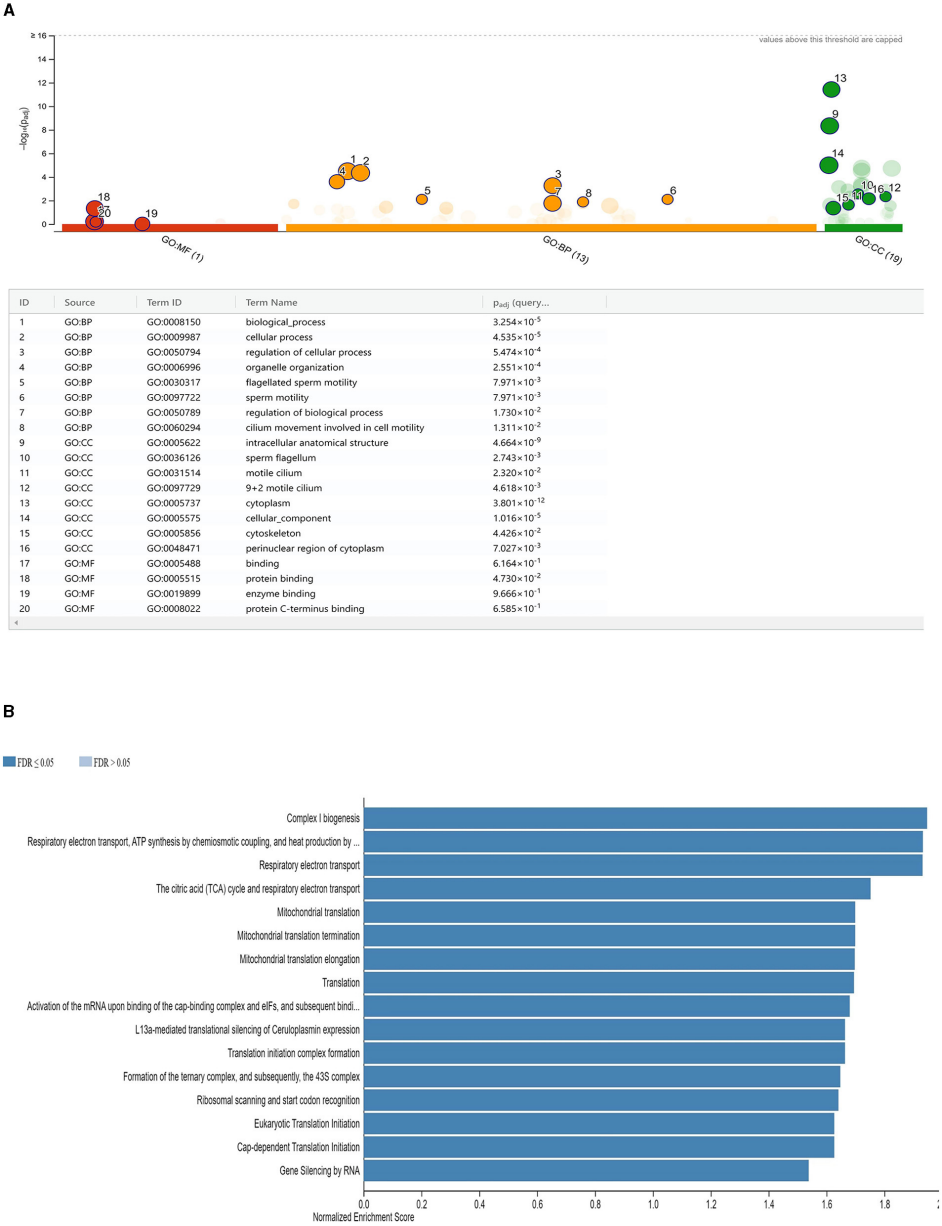
**(A)** Functional enrichment analysis of DE mRNAs in LF and HF sperm of ram. Significantly enriched target gene terms are shown. GO keywords are represented by red color codes, molecular functions by orange ones, biological processes by green ones, and cellular components by green ones (CC). **(B)** Functional enrichment analysis of target genes of *cis- or trans*-regulated by lncRNAs. Illustrated are normalized enrichment scores for specific sets of target genes.

Following a study at the transcriptome level, 275 mRNAs were selected for enrichment analysis due to their differential expression levels (141 upregulated and 134 downregulated). The top eight GO annotations terms in biological process (BP) were significantly enriched in the DE mRNAs, namely, the biological process (GO:0008150), cellular process (GO:0009987), regulation of cellular process (GO:0050794), organelle organization (GO:0006996), flagellated sperm motility (GO:0030317), sperm motility (GO:0097722), and cilium movement involved in cell motility (GO:0060294). In addition, in cellular component, intracellular anatomical structure (GO:0005622), sperm flagellum (GO:0036126), and motile cilium (GO:0031514) were among the top GO terms and binding (GO:0005488), protein binding (GO:0005515), enzyme binding (GO:0019899), and protein C-terminus binding (GO:0008022) in molecular function (MF; Figure 5A). The detailed definition of the GO terms is presented in Supplementary Figure 1. According to the statistics of the pathway enrichment, the top 16 KEGG pathways, including complex I biogenesis, the citric acid (TCA) cycle, respiratory electron transport, respiratory electron transport, mitochondrial translation, and gene silencing by RNA, had higher concentrations of DE mRNAs. *ABHD2, AK1, CABS1, ROPN1, SEPTIN2, SLIRP*, and *TEKT3* genes were commonly enriched for 9 + 2 motile cilium, sperm flagellum, and motile cilium. Moreover, *CABS1, CCDC39, CFAP97D1, ROPN1, SLIRP, TEKT3*, and *TTC12* were commonly enriched in flagellated sperm motility, sperm motility, and cilium movement involved in cell motility (Supplementary Figure 1).

### 3.7 Target genes of cis- or trans-regulated by lncRNAs

To better understand how lncRNAs act in both *cis* and *trans* manner, we predicted their targets. There were 471 mRNAs that were discovered as targets of 10 lncRNAs (five upregulated and five downregulated), using 30 kb as the cutoff (Supplementary Figure 2). According to the GO enrichment study results, 55 significant GO terms were found (corrected *P*-value 0.05). In MF, the top five GO keywords were nucleoside phosphate binding, protein binding, small-molecule binding, binding, and nucleotide binding (Supplementary Figure 3). In BP, negative regulation of the cellular metabolic process, system development, response to chemical stimuli, organonitrogen compound metabolic process, and multicellular organism development were among the top five GO terms and cytoplasm, cell junction, cytosol, nucleoplasm, and synapse in CC (Figure 5B).

## 4 Discussion

The quality of sperm on a cellular level alone is no longer considered to be a reliable predictor of male fertility in selecting superior male prospects in livestock as a source of frozen semen (12, 31). Methods for analyzing the sperm transcriptome, such as measuring messenger RNA levels, have been linked to increased fertility (45). Analysis of the sperm transcriptome has become the primary tool for predicting male fertility potential in the livestock business, due in part to the widespread application of this technology in livestock growth (45, 46). Accordingly, this study demonstrated long noncoding RNA profiles in sperm from rams with HF and LF phenotypes to uncover potential lncRNAs associated with fertility.

The comparative analysis across multiple species indicates a considerable gap in our understanding of the functional roles of lncRNAs within sperm cells despite their prevalence in male germ cell development. Previous studies on cattle, boar, sheep, mice, and humans have collectively identified substantial numbers of potential lncRNAs in sperm cells (46–48). However, the functional annotation and investigation of these lncRNAs have remained limited. We obtained a total of 91,108,174 cDNA libraries, of which 89,252,823 were clean reads from ram spermatozoa. This provides a robust dataset for comparative analysis, especially when considering the consistency with similar studies conducted, such as on human, turkey, and boar (79.8, 84, and about 65.5 million, respectively) spermatozoa (49, 50). Furthermore, the range of unique mapped reads, ranging from 66,623,003 to 78,982,319, indicated a strong alignment of the sequenced data to the reference genome of sheep. The high percentage of over 80% uniquely mapped reads demonstrated a significant mapping consistency comparable to that observed in bull and stallion spermatozoa (32, 36). This consistency across species further confirms the reliability and quality of the obtained sequence data for ram spermatozoa, supporting the validity of results.

In this study on ram fertility, we identified 11,209 sperm lncRNAs, a subset comprising 93 differentially expressed lncRNAs associated with LF and HF phenotypes in ram sperm. Comparatively, in mouse mature spermatozoa, 4,088 novel lncRNA transcripts were identified out of 20,907 known lncRNA transcripts (29), demonstrating the complexity and diversity of these transcripts in different species' sperm cells. Similarly, in human sperm, 27,472 novel lncRNAs were discovered (29), with 19 differential expressions of lncRNA out of 11,561 lncRNA transcripts in mature bull spermatozoa (32). Analysis of goat spermatozoa revealed 655 lncRNA transcripts relevant to spermatogenesis from sequencing of the cDNA library, of which 1% annotated to lncRNAs was similar to ram spermatozoa with a classification of 1.1% (51). We also showed that the type of lincRNA seemed to be closer between ram and goat spermatozoa (34), both revealing a 72.6 and 76.64% annotation to lncRNAs, respectively, which highlights the relevance of these findings across species and their potential implications for understanding male fertility mechanisms.

The GO and KEGG investigations were carried out for associated genes of DE mRNAs and lncRNAs to completely examine the functional roles of mRNAs and lncRNAs in ram sperm fertility. Candidate genes have been identified using this bioinformatics approach, and they were related to male reproductive biology. Our findings demonstrated that 275 mRNA transcripts were enriched for the biological process, cellular component, and molecular function GO characterizations. It has become apparent that several metabolic pathways and regulatory mechanisms have crucial roles in fertility, as related to sperm motility. We demonstrated that *ABHD2, AK1, CABS1, ROPN1, SEPTIN2, SLIRP*, and *TEKT3* genes were commonly enriched for the 9 + 2 motile cilium, sperm flagellum, and motile cilium. Of these, *ABHD2*, an isolated molecule from sperm tails, is needed to activate sperm (52). *AK1* is an enzyme that is often responsible for cellular energy balance. It is detected in the flagella of murine and bovine sperm, which suggests that it is involved in sperm motility and is also demonstrated to be directly associated with bull fertility (53–55). Moreover, *CABS1, CCDC39, CFAP97D1, ROPN1, SLIRP, TEKT3*, and *TTC12* were commonly enriched in flagellated sperm motility, sperm motility, and cilium movement involved in cell motility. As a Ca^2+^ storage protein in mature sperm, *CABS1* is a crucial factor in the regulation of calcium signaling and has been shown to preserve sperm flagella structure (56, 57). *ROPN1*, implicated in fibrous sheath integrity and sperm motility, is engaged in PKA-dependent signaling for spermatozoa capacitation; therefore, mutation and lack of its expression in murine sperm cells cause impaired fertility (58, 59). We showed that differentially abundant protein profiles of sperm from rams with contrasting fertility phenotypes were associated with metabolic energy generation by sperm cells along with the motility signaling pathway (4). Our results are consistent with results from the previous study that mRNA–lncRNA interaction seemed to regulate signaling pathways for functional motility.

The primary potential roles of lncRNAs are to control the expression of neighboring protein-coding genes through both *cis* and *trans* manners, integrating transcriptional coactivation or repression. Consequently, we conducted an in-depth analysis and determined the mRNAs situated within the 30 kilobase (kb) threshold upstream and downstream regions of the differentially expressed lncRNAs. We then employed GO and KEGG analyses on the target genes to determine the undertaken lncRNAs. We found that lncRNAs with different levels of expression are involved in a number of important biological processes. We demonstrated that the identified lncRNA TCONS_00136350 may regulate the differentially expressed coding gene ADAM metallopeptidase domain 32 (*ADAM32*). *ADAM32* is a member of the disintegrin family of membrane-anchored proteins and is detected on the surface of mature sperm (60). *ADAM32* has been demonstrated to be implicated in sperm–egg plasma membrane interaction, and (61) its expression levels are correlated with the quality of human sperm (62). Although *ADAM32* is not required for normal male fertility (63), we showed that it was identified in the top 10 differentially abundant proteins in ram sperm (4). We also discovered that differentially expressed lncRNA TCONS_00035618 modulated enolase 1 (*ENO1*), the protein expression of which was higher in increased fertility of ram sperm (4). *ENO1* plays an important role in the process of metabolism of monocarboxylic acids, and it is a component in the pathway that leads to glycolysis and gluconeogenesis (KEGG:00010) associated with bull fertility (64), and its lower expression levels lead to low motility (65). In line with our study and of others, lncRNAs are potentially important for the coactivation/repression of target genes that regulate sperm fertility and motility.

## 5 Conclusions

Even though several potential lncRNAs have already been identified through cutting-edge technology and accessible lncRNA annotation tools, functional annotation of lncRNAs in sperm biology is still in its infancy and holds great promise. In this study, differentially expressed lncRNAs in ram sperm were ascertained along with their associations with low *vs*. high fertility phenotypes. These findings are important because they help advance both the fundamental science of mammalian male gamete biology and applied science that may provide practical value for potential male fertility markers.

## Data availability statement

The datasets generated and/or analyzed during the current study are available in the EMBL-EBI HYPERLINK http://www.ebi.ac.uk/biostudies/studies/S-BSST1231 accessible at this accession number “S-BSST1231”. The data encompass all raw relevant data.

## Ethics statement

The animal study was approved by Bahri-Dagdas, Research Center Ethical Committee, Turkey (Number: 22.12.2016/58). The study was conducted in accordance with the local legislation and institutional requirements.

## Author contributions

MH: Conceptualization, Data curation, Formal analysis, Funding acquisition, Investigation, Methodology, Project administration, Resources, Supervision, Validation, Visualization, Writing – original draft, Writing – review & editing. AK: Conceptualization, Formal analysis, Investigation, Methodology, Resources, Supervision, Validation, Writing – original draft, Writing – review & editing. EM: Conceptualization, Data curation, Formal analysis, Investigation, Methodology, Project administration, Resources, Supervision, Validation, Visualization, Writing – original draft, Writing – review & editing.

## Supplementary material

Supplementary Figure 1 Distribution of lncRNA on chromosomes. **(A)** Distribution of lncRNAs in low fertility (LF) ram group. **(B)** Distribution of lncRNAs in high fertility (HF) ram group.

Supplementary Figure 2 Identification pipeline for lncRNAs Identification pipeline for lncRNAs. Each step is documented in detail in the Methods section.

Supplementary Figure 3 Functional enrichment analysis of target genes of Cis- or Trans-regulated by lncRNAs. Illustrated are normalized enrichment scores for specific sets of target genes. Significantly enriched target gene terms were shown. GO keywords are represented by red color codes, molecular functions by orange ones, biological processes by green ones, and cellular components by green ones (CC).
