# Supplementary figures and images for: Sperm long non-coding RNAs as markers for ram fertility
Source: Front Vet Sci. 2024 May 10;11:1337939. doi: 10.3389/fvets.2024.1337939 (PMC11117017; doi:10.3389/fvets.2024.1337939)

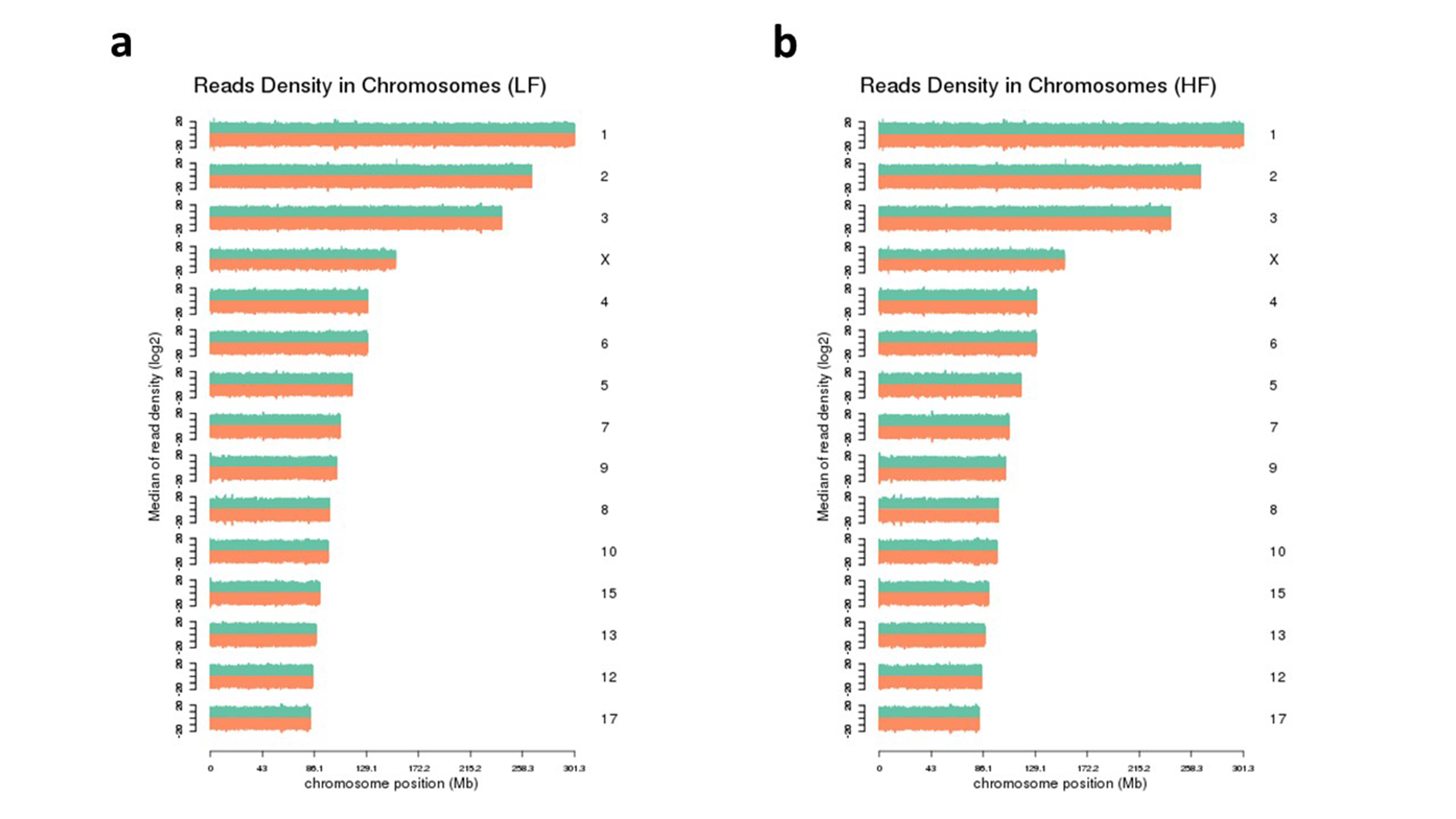

Supplement: Supplementary Figure 1 — Distribution of lncRNA on chromosomes. (A) Distribution of lncRNAs in low fertility (LF) ram group. (B) Distribution of lncRNAs in high fertility (HF) ram group. [file Image_1.JPEG]

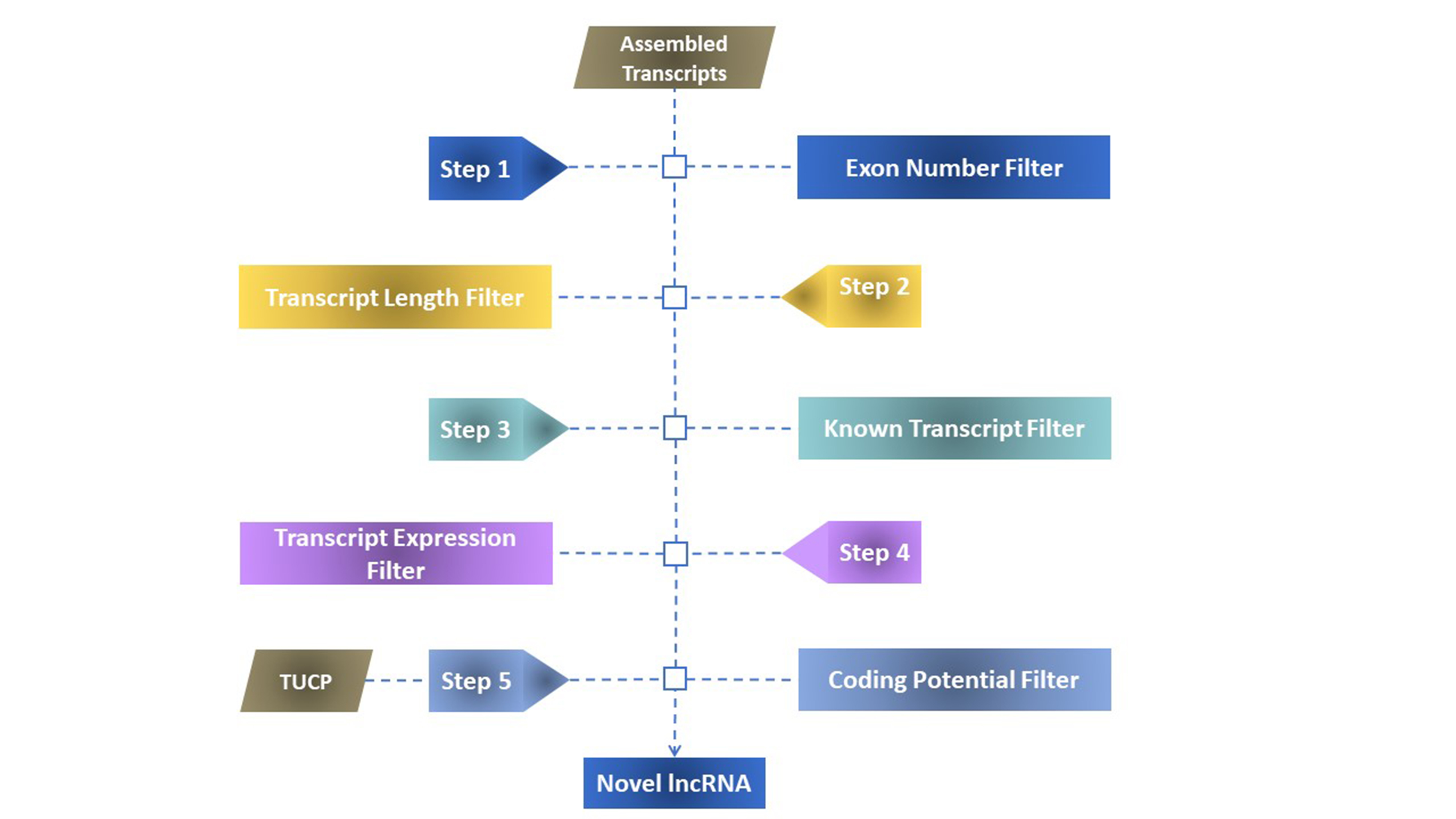

Supplement: Supplementary Figure 2 — Identification pipeline for lncRNAs Identification pipeline for lncRNAs. Each step is documented in detail in the Methods section. [file Image_2.JPEG]
